# The short-term effects of air pollutants on respiratory disease mortality in Wuhan, China: comparison of time-series and case-crossover analyses

**DOI:** 10.1038/srep40482

**Published:** 2017-01-13

**Authors:** Meng Ren, Na Li, Zhan Wang, Yisi Liu, Xi Chen, Yuanyuan Chu, Xiangyu Li, Zhongmin Zhu, Liqiao Tian, Hao Xiang

**Affiliations:** 1 Department of Epidemiology and Biostatistics, School of Public Health, Wuhan University, 115# Donghu Road, Wuhan, 430071, China; 2 Duke Kunshan University, 8# Duke Avenue, Kunshan, 215316, China; 3 State Key Laboratory of Information Engineering in Surveying, Mapping and Remote Sensing, Wuhan University, Luoyu Road 129, Wuhan 430079, China; 4 College of Information Science and Engineering, Wuchang Shouyi University, Wuhan 430064, China

## Abstract

Few studies have compared different methods when exploring the short-term effects of air pollutants on respiratory disease mortality in Wuhan, China. This study assesses the association between air pollutants and respiratory disease mortality with both time-series and time-stratified–case-crossover designs. The generalized additive model (GAM) and the conditional logistic regression model were used to assess the short-term effects of air pollutants on respiratory disease mortality. Stratified analyses were performed by age, sex, and diseases. A 10 μg/m^3^ increment in SO_2_ level was associated with an increase in relative risk for all respiratory disease mortality of 2.4% and 1.9% in the case-crossover and time-series analyses in single pollutant models, respectively. Strong evidence of an association between NO_2_ and daily respiratory disease mortality among men or people older than 65 years was found in the case-crossover study. There was a positive association between air pollutants and respiratory disease mortality in Wuhan, China. Both time-series and case-crossover analyses consistently reveal the association between three air pollutants and respiratory disease mortality. The estimates of association between air pollution and respiratory disease mortality from the case–crossover analysis displayed greater variation than that from the time-series analysis.

The adverse effect of air pollution on health has become a crucial research area in the last decade^1^,^2^,^3^. Extensive clinical, epidemiological, and toxicological studies have provided evidence of relationships between exposure to ambient air contaminants and human health^4^,^5^,^6^,^7^,^8^,^9^,^10^,^11^. The short-term effects of air pollution have mainly been demonstrated by increases in the mortality and morbidity of respiratory diseases. Thus, the effect of air pollution on deaths from respiratory diseases has aroused great concern among epidemiological researchers.

Time-series and case-crossover analyses are two common epidemiology methods that are frequently used to assess the health effects of air pollution. Time-series analysis, which allows for adjustment of time-invariant confounders, examines the same population repeatedly under various exposure conditions^12^. Additionally, the introduction of generalized additive models (GAMs) has enabled adjustment for the confounding effects of trends and seasonality, which has boosted research on associations between air pollution and health^13^. However, time-series analysis has been criticized for its complexity and the possibility of producing incorrect standard errors upon correlation between nonlinear functions^14^,^15^. The case-crossover design compares exposure during a case day when events occurred (e.g., deaths) with exposures in nearby control days to examine whether the events are associated with a particular exposure^16^. It has the advantage of controlling time-invariant individual confounders, and by matching case and control days within a short interval (usually within 28 days or a month), time-variant confounders are also removed^17^.

The city of Wuhan is confronted with a serious air pollution problem. Much attention has been focused on this fast-developing city with rapid industrialization, urbanization, and motorization, and attempts are being made to assess the impact of air pollution. This study was undertaken to investigate the association between air pollutants and daily respiratory disease mortality in Wuhan from 2007 to 2009 by using both time-series and case-crossover designs. Since time-series and case-crossover analyses are viewed as competing methods in air pollution epidemiology, the study also examined whether these methods produced equivalent risk estimates.

## Materials and Methods

### Data Collection

Data regarding average daily air mass index (Air Quality IndexAQI)^3^of particulate matter less than 10 μm in aerodynamic diameter (PM_10_), sulfur dioxide (SO_2_), and nitrogen dioxide (NO_2_) were obtained from Wuhan Municipal Environmental Protection Bureau from 2007 to 2009 in Wuhan. This information was converted into a daily average concentration of atmospheric pollutants based on the “Ambient Air Quality Standard.” (GB3095-2012) (http://kjs.mep.gov.cn/hjbhbz/bzwb/dqhjbh-/dqhjzlbz/201203/t20120302_224165.htm) The daily concentration of pollutants was represented by the mean value of nine national fixed-site stations. These monitoring stations are required to be a certain distance away from pollution sources according to the national technical guidelines.

Meteorological data were obtained for the same period from the Wuhan Meteorological Monitoring Database, including daily average temperature, barometric pressure, and relative humidity.

Mortality data were collected from the death registration system in Hubei Provincial Center for Disease Control and Prevention from January 1, 2007, to December 31, 2009. Data on deaths from respiratory diseases were classified according to the International Classification of Disease, 10th revision (ICD10: J00-J99). In this study, three common respiratory diseases (pneumonia (J18), chronic obstructive pulmonary disease(COPD, J40-J44 and J47), and asthma (J45)) were analyzed.

### Data Analysis

We used GAM to perform time-series analysis and conditional logistic regression modeling of the case-crossover design.

Many factors affect respiratory disease mortality such as living habits, socioeconomic status, and so on. However, most of these factors varied with time or exhibited a seasonal trend. In GAM, we used a natural spline approach to control the factors that were related to time or seasonal trends and varied the degree of freedom per year for time to identify a best approach to control for these factors. If the relative risk did not change beyond the present value, then we regarded it as the “best” degree of freedom^18^. A polynomial distributed lag model was used to examine exposure to air pollution per day, to identify which of the days had the strongest association with respiratory disease mortality, and to examine the lag effect for 2 days with 4 degrees of freedom. Given time-series data on pollution levels, mortality count, and other variables, we used the following statistical model:


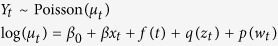



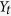
 is the respiratory disease mortality count for day *t*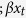 represents the variables such as PM_10_, SO_2,_ or NO_2_ concentration for day *t*; 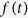 is a smooth function of the time variable *t*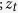 represents temperature count for day *t;* 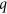 is a (smooth) function of temperature; 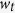
represents relative humidity count for day *t;* and 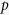 is a (smooth) function of humidity.

In the conditional logistic regression model, we weighted the number of daily deaths due to respiratory diseases. When calculating the value of the odds ratio (OR) in the regression model, meteorological factors including daily average temperature, barometric pressure, and relative humidity were introduced as concomitant variables and the lag effects of air pollutant increments were considered.

Case-crossover study is a type of observational research methods, commonly used for studying the short-term or transient effects of exposures or risk factors. There were two parts in the study, including cases and controls, which were from the same individual. “Part of cases” referred to a dangerous period before the occurrence of the disease or event. “Control part” was a specific period, with the exception of the dangerous period. In a case-crossover study, cases serve as their own controls, and therefore, the design eliminates confounding by stable individual characteristics such as age, sex, health status, and behavioral factors^19^. This study analyzed the effect of air pollution in the same death case, during the dangerous period and a specific period before it, to match case days with control days in a short interval of 28 days.

For each pollutant, the relative risks (RRs) for time-series analysis, ORs for time-stratified case-crossover analysis, and 95% confidence intervals (CIs) were calculated. All statistical tests were two-sided, and P values <0.05 were considered statistically significant. R software (version 2.12.2, R Foundation for Statistical Computing, Vienna, Austria) and the mixed GAM computation vehicle package were applied to fit, in both time-series model and GAM. SAS 9.2 (SAS system for Windows, release 9.2, SAS Institute, Cary, NC) was applied in the time-stratified case-crossover analysis.

## Results

### Air Pollutants and Mortality Data

Table 1 summarizes the demographic characteristics of the study population and respiratory deaths. From January 1, 2007, to December 31, 2009, there were a total of 19,948 deaths (excluding accidents and injuries), of which 2,120 were due to respiratory diseases. The results show that mortality differed based on sex and age, and a greater number of deaths were found in men and subjects older than 65 years.

Table 2 shows the distribution of air pollution and meteorological measurements in Wuhan. Over a 3-year period, the mean daily pollutant levels were 113.81 μg/m^3^ for PM_10_, 54.40 μg/m^3^ for NO_2_ and 52.04 μg/m^3^ for SO_2_ (Table 2).

The Spearman’s correlation coefficients among the air pollutants are presented in Table 3. There was a certain degree of correlation among the pollutants, especially between PM_10_ and NO_2_ (r = 0.764), and all 3 air pollutants were negatively correlated with temperature and humidity. As described in our previous article, the long-term and smoothed-trend of daily average concentrations showed a strong seasonal pattern, with higher concentrations occurring during spring and winter, and lower ones occurring during summer and fall^20^.

### Associations Between Air Pollutants (PM_10_, NO_2_, SO_2_) and Respiratory Disease Deaths

Table 4 shows the effect of air pollutants on overall daily mortality in single-pollutant models, and similar results were found in time-series and case-crossover analyses. Adjusting for temperature and relative humidity, results of both the GAM and conditional logistic regression model showed an association between SO_2_ and respiratory disease mortality. A 10 μg/m^3^ increment in SO_2_ on the same day and over a 24-hour period were associated with overall respiratory disease mortality OR of 1.9% (0.1%, 3.7%) and 2.4% (0.7%, 4.1%), respectively. In addition, RR of respiratory disease mortality over a 24-hour period was 1.9% (0.2%, 3.6%) in the time-series model. However, no statistical associations were found between PM_10_, NO_2,_ and overall respiratory disease mortality in both models.

### Associations Between Air Pollutants (PM_10_, SO_2_, NO_2_) and Respiratory Disease Deaths among Different Groups of People

Table 5 illustrates the effect estimate of air pollutants on respiratory disease mortality among different groups of people. After adjusting the temperature and relative humidity, an association between a 3-day average SO_2_ and respiratory diseases deaths was found only for men in both models. For a 10 μg/m^3^ increase in 3-day average SO_2_, RR was 2.7% (0.1%, 5.4%) and OR was 4.3% (−1.5%, 7.1%). In addition, an association between NO_2_ on lag2 days and respiratory disease deaths among men were demonstrated in both models. In addition, SO_2_ on lag1 day and NO_2_ on lag2 days were also significantly associated with respiratory disease deaths among people older than 65 years old when using case-crossover analysis. Except for these, no statistically significant associations were found among other groups of people.

### Associations Between Pollutants (PM_10_, SO_2_, NO_2_) and Different Types of Respiratory Diseases

Table 6 shows the associations between air pollutants and three different respiratory diseases. Both time-series and case-crossover analyses showed that only pneumonia was associated with increasing PM_10_ concentration for one day lagged. The results suggest no evidence for effects of PM_10_, NO_2,_ and SO_2_ on chronic obstructive pulmonary disease and asthma mortality.

### Associations Between Pollutants (PM_10_, SO_2_, NO_2_) and Respiratory Diseases in Multiple Pollutants Models

**Figure 1:**
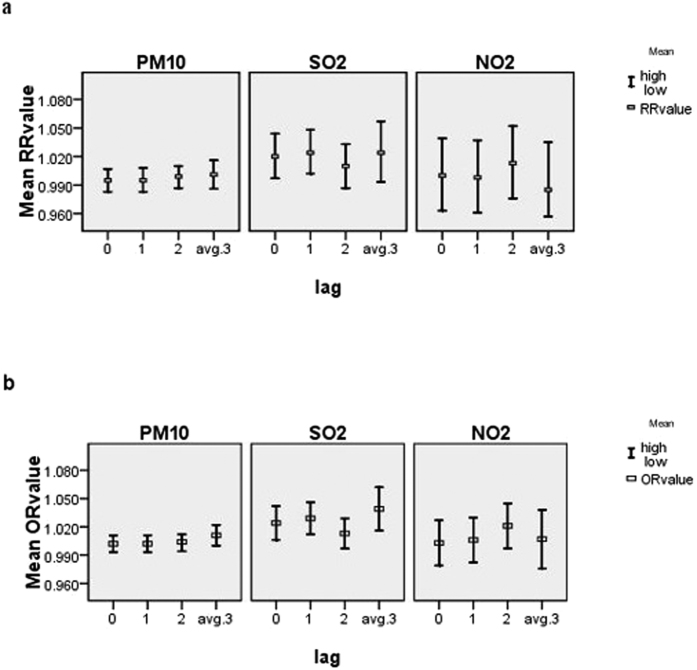
(**a**) Effects of air pollutants on overall respiratory mortality in multiple-pollutants models in Time-series. (**b**) Effects of air pollutants on overall respiratory mortality in multiple-pollutants models in Case-crossover.

Table 4 and Table S1 illustrate the effect estimates of air pollutants on overall respiratory disease mortality and respiratory mortality among different people and types of diseases in multiple-pollutants models. Figure 1a and b showed the effects of air pollutants on overall respiratory mortality in multiple-pollutants models in time-series and case-crossover analysis, respectively. In three pollutant models, the effect of SO_2_ on overall mortality remained even after the inclusion of PM_10_ and NO_2_ in both time-series and case-crossover analyses. For pneumonia, the effect of PM_10_ remained and the values of OR and RR were higher than that in single pollutant model. Besides, effects of SO_2_ and NO_2_ on pneumonia also became significant when other two pollutants were added in the models in time-series analysis. We observed effects of PM_10_ and SO_2_ on asthma in multiple pollutants models in time-series analysis, which was not found in single pollutant models.

## Discussion

### Characteristics of Air Pollutants in Wuhan

Wuhan is faced with increasingly severe air pollution, and the air quality of Wuhan ranks in the middle of the cities in China, with its fast development in recent years. As an important industrial and transportation hub of China, the main sources of air pollutants in Wuhan come from vehicle exhaust, dining fumes, coal-fired heating, and industrial emissions. The major industry of Wuhan includes optoelectronic communications, automobile manufacturing, steel manufacturing, chemical, and power generation industries.

As shown in our previous study, the daily average concentrations of three major air pollutants in Wuhan (PM_10_, NO_2_, and SO_2_) were higher in spring and winter than in summer and autumn. Certain meteorological factors such as temperature and humidity had different impacts on PM_10_, NO_2_, and SO_2_, which were similar to the findings by Pei regarding the spatial and temporal distribution of air pollutants in Wuhan, during 2007–2011^21^. Possible explanations for this type of change were given in our previous study^20^.

### Associations Between Air Pollutants and Respiratory Diseases

Results of this study showed that among three major air pollutants analyzed in Wuhan, only SO_2_ had the most significant effect on respiratory disease mortality in both time-series and case-crossover analyses. A 10 μg/m^3^ increment of SO_2_ levels was associated with 2.4% and 1.9% increase of relative risk of overall respiratory disease mortality in case-crossover and time-series analyses, respectively. The research conducted in 12 European cities by Kassiani *et al*. showed that respiratory disease mortality increased by 5.0% per 50 μg/m^3^ increment of SO_2_^22^. In Beijing, Liu *et al*. found that daily respiratory disease mortality increased with SO_2_ by 0.88% (0.34%～1.41%) per 10 μg/m^3^ increment^23^. Research focused on the relationship between atmospheric gaseous pollutants and daily mortality conducted in Shanghai showed that respiratory disease mortality increased with SO_2_ by 1.71% per 10 μg/m^3^ increment^24^. Thus, our results are consistent with previous research findings on the health effect of SO_2_, which were estimated in both methods. In this study, no significant associations were found between either PM_10_ or NO_2_ and overall respiratory disease mortality, regardless of which method was used, which is inconsistent with findings from many other studies^25^,^26^,^27^,^28^. A similar study conducted by Liu *et al*. in Wuhan from January 2005 to December 2006, using case-crossover analysis, found that all three major air pollutants (PM_10_, NO_2_, and SO_2_) were significantly associated with daily respiratory disease mortality when adjusted for different confounders^29^. This may be related to different study periods as climate conditions and types of air pollution in a city would vary with time. The study conducted by Zhang *et al*. in Chaoyang District, Beijing, also showed that both SO_2_ and NO_2_ were significantly associated with daily overall respiratory disease mortality, whereas there was no significant association for PM_10_ 30. Such inconsistencies might be related to the age of the exposed population, sensitivity of the population to air pollution, types of urban pollutants, and pollution levels, as well as many other cultural and social demographic factors^31^. Moreover, an Italian study showed that every interquartile increase in NO_2_ was associated with a 2.5% (0.9%～4.2%) increase in respiratory disease hospital admissions and a 0.4% (−1 3%～2.2%) increase for every interquartile increase in PM_10_, but the impact of SO_2_ on respiratory disease mortality was not obvious^32^. Long-term high levels of air pollution might increase the adaptability of the population. In addition, the main components of PM_10_ in Wuhan may be associated with lower toxicity than those found in developed countries, where the components of PM_10_ are mainly from vehicle exhaust and associated with higher toxicity in humans^33^.

In a stratified analysis, the results estimated from the two methods showed some tiny differences. The effects of SO_2_ and NO_2_ on respiratory disease mortality in men were significant in both case-crossover and time-series analysis. In women, neither method showed a significant association with the three major air pollutants. Tao *et al*., using a semi-parametric generalized additive model, conducted a study in Lanzhou and came to a similar conclusion: the effects of atmospheric pollutants on respiratory diseases were more obvious in men than in women^34^. Anderson pointed out that this kind of difference may be caused by a higher proportion of men in outdoor jobs and weaker self-protection awareness among men. In addition, unhealthy lifestyle choices such as smoking and drinking are more common in men than in women, and therefore, men may be more susceptible to external pollutant-induced respiratory diseases^35^.

In the analysis of effects on different types of respiratory disease, pneumonia was significantly associated with PM_10_ in both time-series and case-crossover analysis whenever SO_2_ and NO_2_ are included or not in the model. The association of PM_10_ and pneumonia mortality was enhanced after adjusting for the influences of NO_2_ and SO_2_, which suggested possible confounding effects. Moreover, after adjusting for the NO_2_ and SO_2_, the association of PM_10_ and asthma became significant in time-series analysis. The effects of PM_10_ on respiratory disease mortality supported the previous findings that the effects of particulate matter exacerbated the inflammation in immunocompromised individuals. The mechanism is that particulate matter acts as an immunosuppressive agent and disrupts normal pulmonary antimicrobial defenses, which is supported by animal models and epidemiological studies^36^,^37^. SO_2_ was found to have the most significant effect on overall respiratory disease mortality in our study. However, it was only significantly associated with pneumonia at lag1 day in multiple pollutant models and only in time-series analysis. Consistently, a time-series study conducted in Beijing, on the relationship between SO_2_ and daily mortality from 2006 to 2009, illustrated that every 10 μg/m3 increment in SO_2_ was associated with 0.88% of increment in daily respiratory disease mortality and 1.43% increment in pneumonia mortality^23^. The levels of SO_2_ were positively associated with daily respiratory disease mortality and daily pneumonia mortality at lag0 day and lag1 day, respectively. These results suggest that levels of SO_2_ have acute effects on respiratory disease mortality and pneumonia mortality and there are no obvious lag effects. Therefore, timely preventive measures are supposed to be applied in reducing the health effects of air pollution in Wuhan, when extreme air pollution days appear. This will depend on better awareness of risks of air pollution by governments, timely responses by the health alert system, public health professionals and precautionary measures by the general public.

Interaction effects of air pollutants have been one of difficulties in the study of atmospheric environmental epidemiology. In general, it is hard to distinguish independent effects of a single air pollutant because of different characteristics and exposure methods of every air pollutant. As shown in the Table 3, each kind of air pollutant is closely correlated with each other. Thus, we think that single pollutant models estimated effects of air pollution on mortality better than multiple pollutant models in this study.

### Comparison of Case-crossover and Time-series Analyses

In our study, the results of case-crossover analysis were compared with those of the time series analysis and found to be statistically significant, with the exception of a few tiny differences in the stratified analyses. Among the three air pollutants, only SO_2_ was statistically associated with overall respiratory disease mortality in the two different analyses. Compared to the OR value between SO_2_ and respiratory disease mortality in the case-crossover analysis, the value of RR in the time-series analysis was a little lower, but it had a narrower confidence interval. In the analysis of effects on different types of respiratory disease, the case-crossover analysis showed that increments of SO_2_ and NO_2_ were significantly associated with respiratory disease mortality among people older than 65 years, but the case-crossover analysis did not share these findings. Similar studies have made compared the two methods based on residual checking. The results usually showed that time-series analysis performed better than time-stratified–case-crossover analysis, as there was far less autocorrelation in the residuals in the time-series analysis^16^. This result could be caused by the case-crossover design assuming a step-like seasonal change^38^, whereas the time series assumed a smoothly changing seasonal pattern. Figueiras *et al*. pointed out that if the impact of autocorrelation on estimates had been successfully controlled for, case-crossover analysis could be a good alternative to time-series analysis using Poisson regression^39^. However, the effects of unmeasured confounding variables that do not vary over time can be controlled by the case-crossover design.

In general, consistent results were found by using either the time-series or time-stratified–case-crossover analysis. Our findings support and extend previous studies, demonstrating that both time-series and time-stratified–case-crossover methods can be used to estimate the event or episode-related health effects and produce robust and comparable results.

### Strengths and Limitations

The study had two key strengths. First, the sources of data ensured their reliability and validity. The meteorological data were collected from Wuhan Meteorological Database and the mortality data were collected from death registration system in Hubei Provincial Center for Disease Control and Prevention, which provided relatively accurate information. Additionally, the short-term effect of ambient air pollutants was assessed by application and comparison of two different methods, which made the results more convincing. The second strength was that some time-invariant variables, such as sex, age, and family history, did not act as confounders because the case-crossover design controlled confounders by the design rather than the models^40^,^41^. In addition, we could avoid possible confounding from the effects of day of the week by controlling the same weekday as the onset day^42^,^43^.

We do acknowledge that there were several limitations in our study. First, like other similar studies in this field, we obtained available data on ambient air pollution and weather conditions from fixed monitoring stations and assumed that they adequately represented the exposure for the population, which would inevitably lead to some measurement errors for exposure. Such errors often lead to underestimating the health effects of air pollution. In addition, we were not able to take into consideration indoor air pollution because of the unavailability of data, which may also have affected the personal exposure levels and overestimated the observed associations between ambient air pollution and mortality. Moreover, there is no well-accepted individual exposure assessment method in epidemiological studies on air pollution. Finally, as for indicators of air pollution, PM_2.5_ is associated with a higher health risk than PM_10_ and is a better indicator of air pollution^44^. Since the daily concentration of PM_2.5_ for the period was not available, this was a limitation when assessing the health effects of particulate matter; in the future, more research about health effects of PM_2.5_ needs to be conducted.

## Conclusion

Results of this study indicated a positive association between air pollutants and respiratory disease mortality in Wuhan, China. Our results confirm previous studies on the adverse effects of long-term exposure to air pollution. This association further contributes to the evidence that exposure to ambient air pollution is a significantly hazardous factor to respiratory health.

## Additional Information

**How to cite this article:** Ren, M. *et al*. The short-term effects of air pollutants on respiratory disease mortality in Wuhan, China: comparison of time-series and case-crossover analyses. *Sci. Rep.* **7**, 40482; doi: 10.1038/srep40482 (2017).

**Publisher's note:** Springer Nature remains neutral with regard to jurisdictional claims in published maps and institutional affiliations.

## Supplementary Material

Supplementary Table S1

## Figures and Tables

**Table 1:** Description of characteristics of all subjects collected during 2007–2009.

characteristics		2007 (%)	2008 (%)	2009 (%)	Total (%)
Respiratory diseases		573 (27.0)	597 (28.2)	950 (44.8)	2120 (100)
Age	＜65	86 (15.0)	65 (10.9)	125 (13.2)	276 (13.0)
	≥65	487 (85.0)	532 (89.1)	825 (86.8)	1844 (87.0)
Gender	Male	363 (63.4)	399 (66.8)	587 (61.8)	1349 (63.6)
	Female	210(36.6)	198 (33.2)	363 (38.2)	771 (36.4)
Classification	pneumonia	35 (6.1)	45 (7.5)	62 (6.5)	142 (6.7)
	COPD	188 (32.8)	253 (42.4)	392 (41.3)	833 (39.3)
	Asthma	146 (25.5)	128 (21.5)	207 (21.8)	481 (22.7)
	Others	204 (35.6)	171 (28.6)	289 (30.4)	664 (31.3)

**Table 2:** Descriptive Statistics for PM_10_, SO_2_, NO_2_, Temperature and Relative Humidity in Wuhan, 2007–2009.

	Mean	Standard Deviation	IQR	Min	Q1	Q2	Q3	Max
PM10 (μg/m^3^)	113.81	54.35	72.00	18.00	74.00	106.00	146.00	567.00
SO_2_ (μg/m^3^)	52.04	30.43	35	8.00	31.00	45.00	66.00	267.00
NO_2_ (μg/m^3^)	54.40	21.63	27.20	19.20	38.40	49.60	65.60	153.60
Temperature (°C)	18.06	9.47	16.30	−2.7	9.8	19.45	26.1	35.30
Relative humidity (%)	70	13	18	21	61	70	79	97

**Table 3:** Spearman coefficient of meteorological factor and air pollutants in Wuhan, 2007–2009.

	PM10	SO2	NO2	Avg. Temperature	Avg. Humidity
PM10	1	—	—	—	—
SO2	0.715^b^	1	—	—	—
NO2	0.764^b^	0.752^b^	1	—	—
Avg. Temperature	−0.204^b^	−0.391^b^	−0.381^b^	1	—
Avg. Humidity	−0.239^b^	−0.283^b^	−0.120^b^	−0.188^b^	1

b: P < 0.01.

**Table 4:** Associations between air pollutants and overall respiratory disease mortality.

	Single Pollutant Model		Multiple Pollutants Model	
	Time series	Case-crossover	Time series	Case-crossover
	RR(95%CI)	OR(95%CI)	RR(95%CI)	OR(95%CI)
PM10				
Lag0	1.000 (0.991,1.010)	1.007 (0.998,1.016)	0.995 (0.983,1.007)	1.002 (0.993,1.011)
Lag1	1.002 (0.993,1.011)	1.009 (1.000, 1.018)	0.995 (0.983,1.008)	1.002 (0.993,1.011)
Lag2	1.005 (0.996,1.013)	1.010 (1.002, 1.018)	0.999 (0.987,1.010)	1.004 (0.994,1.012)
Ave.3 days	1.005 (0.994,1.016)	1.015 (1.004, 1.026)	1.001 (0.986,1.016)	1.011 (1.000,1.022)
SO_2_				
Lag0	1.015 (0.998,1.032)	**1.019**^**a**^ (1.001, 1.037)	1.020 (0.997,1.044)	1.024 (1.006,1.042)
Lag1	**1.019**^**a**^ (1.002,1.036)	**1.024**^**a**^ (1.007, 1.041)	**1.024**^**a**^ (**1.002,1.048)**	**1.029**^**a**^ (**1.012,1.046)**
Lag2	1.015 (0.998,1.031)	1.018 (1.002, 1.034)	1.010 (0.987,1.033)	1.013 (0.997,1.029)
Ave.3 days	1.018 (0.997,1.039)	1.033 (1.010, 1.056)	1.024 (0.993,1.057)	1.039 (1.016,1.062)
NO_2_				
Lag0	1.010 (0.985,1.037)	1.013 (0.989, 1.037)	1.000 (0.963,1.039)	1.003 (0.979,1.027)
Lag1	1.015 (0.990,1.041)	1.023 (0.999, 1.047)	0.998 (0.961,1.037)	1.006 (0.982,1.030)
Lag2	1.021 (0.997,1.047)	1.029 (1.005, 1.053)	1.013 (0.976,1.052)	1.021 (0.997,1.045)
Ave.3 days	1.013 (0.982,1.045)	1.035 (1.004, 1.066)	0.985 (0.937,1.035)	1.007 (0.976,1.038)

a: The RRs or ORs are statistically significant (P-value < 0.05).

**Table 5:** Associations between air pollutants and respiratory disease mortality among different groups of people using single pollutant models.

	Males		Females		＜65 years old		≥65 years old	
	Time series	Case-crossover	Time series	Case-crossover	Time series	Case-crossover	Time series	Case-crossover
	RR (95%CI)	OR (95%CI)	RR (95%CI)	OR (95%CI)	RR(95%CI)	OR (95%CI)	RR (95%CI)	OR (95%CI)
PM10								
Lag0	1.003 (0.992,1.015)	1.009 (0.998,1.020)	1.001 (0.986,1.016)	1.006 (0.991,1.021)	0.998 (0.983,1.014)	1.004 (0.981,1.027)	0.998 (0.987,1.010)	1.008 (0.998,1.018)
Lag1	1.004 (0.993,1.015)	1.010 (0.999,1.021)	1.000 (0.985,1.015)	1.010 (0.996,1.024)	1.001 (0.986,1.016)	0.993 (0.972,1.014)	1.000 (0.989,1.011)	1.012 (1.002,1.022)
Lag2	1.010 (1.000,1.021)	0.975 (0.965,0.985)	0.994 (0.979,1.008)	1.003 (0.988,1.018)	1.001 (0.987,1.016)	1.000 (0.981,1.019)	1.003 (0.992,1.014)	1.010 (1.001,1.019)
Ave.3days	1.010 (0.996,1.023)	1.017 (1.003,1.031)	0.999 (0.981,1.017)	1.004 (0.985,1.023)	0.998 (0.980,1.015)	0.992 (0.965,1.019)	1.005 (0.992,1.019)	1.019 (1.006,1.032)
SO2								
Lag0	1.011 (0.989,1.003)	**1.020**^**a**^ (**0.998,1.042)**	1.021 (0.994,1.049)	1.017 (0.988,1.046)	1.016 (0.992,1.040)	1.026 (0.982,1.070)	1.005 (0.980,1.031)	1.014 (0.995,1.033)
Lag1	**1.022**^**a**^ (**1.002,1.044)**	**1.034**^**a**^ (**1.013,1.055)**	1.014 (0.987,1.041)	1.008 (0.981,1.035)	1.013 (0.991,1.037)	0.988 (0.948,1.028)	1.015 (0.991,1.040)	**1.023**^**a**^ (**1.005,1.041)**
Lag2	**1.026**^**a**^ (**1.005,1.046)**	1.028 (1.008,1.048)	0.998 (0.972,1.025)	0.997 (0.971,1.023)	1.003 (0.980,1.026)	0.991 (0.953,1.029)	1.016 (0.993,1.040)	1.018 (1.001,1.035)
Ave.3days	**1.027**^**a**^ (**1.001,1.054**)	**1.043**^**a**^ (**1.015,1.071)**	1.007(0.974,1.041)	1.014 (0.978,1.050)	1.001 (0.973,1.029)	0.994 (0.968,1.020)	1.021 (0.990,1.053)	1.033 (1.008,1.058)
NO2								
Lag0	1.015 (0.983,1.048)	1.024 (1.002,1.046)	0.998 (0.958,1.040)	0.997 (0.957,1.037)	0.994 (0.956,1.033)	1.017 (0.957,1.077)	1.017 (0.984,1.052)	1.015 (0.989,1.041)
Lag1	1.023 (0.991,1.055)	1.037 (1.016,1.058)	0.994 (0.953,1.036)	1.000 (0.961,1.039)	0.998 (0.960,1.037)	0.974 (0.917,1.031)	1.018 (0.985,1.053)	1.031 (1.005,1.057)
Lag2	**1.038**^**a**^**(1.007,1.070)**	**1.047**^**a**^ (**1.027,1.067**)	0.989 (0.950,1.030)	0.996 (0.958,1.034)	1.002 (0.965,1.040)	0.996 (0.940,1.052)	1.020 (0.988,1.054)	**1.028**^**a**^ (**1.002,1.054)**
Ave.3days	1.033 (0.995,1.073)	1.055 (1.027,1.083)	0.977 (0.929,1.027)	0.997 (0.946,1.048)	0.986 (0.943,1.031)	0.983 (0.911,1.055)	1.014 (0.973,1.058)	1.033 (0.999,1.067)

a: The RRs or ORs are statistically significant (P-value <0.05).

**Table 6:** Associations between air pollutants and mortality of different respiratory disease using single pollutant models.

	COPD		Pneumonia		Asthma	
	Time series	Case-crossover	Time series	Case-crossover	Time series	Case-crossover
	RR(95%CI)	OR(95%CI)	RR(95%CI)	OR(95%CI)	RR(95%CI)	OR(95%CI)
PM10						
Lag0	1.000 (0.985,1.015)	1.011 (0.995,1.027)	1.016 (0.982,1.050)	1.023 (0.982,1.064)	1.002 (0.983,1.020)	1.004 (0.982,1.026)
Lag1	0.997 (0.983,1.012)	1.002 (0.986,1.018)	**1.043**^**b**^ (**1.011,1.075)**	**1.048**^**a**^ (**1.012,1.084)**	0.990 (0.972,1.008)	0.987 (0.966,1.008)
Lag2	1.001 (0.988,1.016)	1.004 (0.989,1.019)	1.009 (0.977,1.043)	0.993 (0.958,1.028)	0.992 (0.974,1.010)	0.990 (0.968,1.012)
Ave.3days	1.003 (0.985,1.020)	1.009 (0.989,1.029)	1.031 (0.991,1.072)	1.035 (0.989,1.081)	0.983 (0.961,1.004)	0.992 (0.966,1.018)
SO2						
Lag0	1.010 (0.982,1.039)	1.015 (0.984,1.046)	0.943 (0.871,1.021)	0.951 (0.866,1.036)	1.030 (0.999,1.063)	1.014 (0.973,1.055)
Lag1	1.019 (0.992,1.047)	1.016 (0.985,1.047)	1.060 (0.994,1.131)	1.045 (0.971,1.119)	1.014 (0.983,1.047)	0.999 (0.961,1.037)
Lag2	0.998 (0.972,1.025)	1.000 (0.971,1.029)	1.008 (0.941,1.081)	0.960 (0.890,1.030)	1.020 (0.987,1.053)	0.997 (0.959,1.035)
Ave.3days	1.010 (0.976,1.044)	1.018 (0.980,1.056)	1.034 (0.950,1.126)	0.990 (0.896,1.084)	1.017 (0.978,1.058)	1.009 (0.962,1.056)
NO2						
Lag0	1.017 (0.976,1.060)	1.020 (0.976,1.064)	0.958 (0.864,1.063)	0.945 (0.846,1.044)	1.013 (0.964,1.064)	1.018 (0.961,1.075)
Lag1	1.007 (0.966,1.050)	1.008 (0.966,1.050)	0.990 (0.894,1.097)	0.999 (0.897,1.101)	1.029 (0.980,1.080)	1.020 (0.963,1.077)
Lag2	0.985 (0.947,1.026)	0.997 (0.956,1.038)	0.992 (0.898,1.095)	0.980 (0.883,1.077)	1.032 (0.982,1.084)	1.013 (0.959,1.067)
Ave.3days	0.991 (0.943,1.042)	1.014 (0.961,1.067)	0.973 (0.861,1.100)	0.958 (0.840,1.076)	1.014 (0.958,1.072)	1.028 (0.960,1.096)

a: The RRs or ORs are statistically significant (P-value <0.05). b: The RRs or ORs are statistically significant (P-value <0.01).
