# Supplementary material for: The short-term effects of air pollutants on respiratory disease mortality in Wuhan, China: comparison of time-series and case-crossover analyses
Source: Sci Rep. 2017 Jan 13;7:40482. doi: 10.1038/srep40482 (PMC5234024; doi:10.1038/srep40482)
Supplement: Supplementary Table S1 [file srep40482-s1.pdf]

The short-term effects of air pollutants on respiratory disease mortality in Wuhan, China: comparison of time-series and case-crossover analyses

Meng Ren[a†], Na Li[a†], Zhan Wang[b], Yisi Liu[a], Xi Chen[a], Yuanyuan Chu[a], Xiangyu Li[a], Zhongmin Zhu[c, d], Liqiao Tian[c], Hao Xiang[a*].

a. Department of Epidemiology and Biostatistics, School of Public Health, Wuhan University, 115# Donghu Road, Wuhan, 430071, China;

E-mails: melodyrren@163.com; nali2012@whu.edu.cn; roselewis@sina.com; aries_c_7@163.com; 2014203050033@whu.edu.cn; lxy329880@163.com

b. Duke Kunshan University, 8# Duke Avenue, Kunshan, 215316, China;

E-mail: zhan.wang@dukekunshan.edu.cn

c. State Key Laboratory of Information Engineering in Surveying, Mapping and Remote Sensing, Wuhan University, Luoyu Road 129, Wuhan 430079, China;

Emails: zhongmin.zhu@whu.edu.cn; tianliqiao@whu.edu.cn

d. College of Information Science and Engineering, Wuchang Shouyi University, Wuhan 430064, China

†: These authors contributed equally to this work.

*: Author to whom correspondence should be addressed;

E-Mail:xianghao@whu.edu.cn;

Tel.: +86-27-6875-9118.

Table S1 Odds ratios and relative risks for respiratory diseases mortality for a 10μg/m^3 increase in air pollutants in a three-pollutant model.

		PM10				SO_2				NO_2			
		Lag0	Lag1	Lag2	Ave.3days	Lag0	Lag1	Lag2	Ave.3days	Lag0	Lag1	Lag2	Ave.3days
respiratory diseases	Time series	0.995(0.983,1.007)	0.995(0.983,1.008)	0.999(0.987,1.010)	1.001(0.986,1.016)	1.020(0.997,1.044)	**1.024[a](1.002,1.048)**	1.010(0.987,1.033)	1.024(0.993,1.057)	1.000(0.963,1.039)	0.998(0.961,1.037)	1.013(0.976,1.052)	0.985(0.937,1.035)
	Case-crossover	1.002(0.993,1.011)	1.002(0.993,1.011)	1.004(0.994,1.012)	1.011(1.000,1.022)	1.024(1.006,1.042)	**1.029[a](1.012,1.046)**	1.013(0.997,1.029)	1.039(1.016,1.062)	1.003(0.979,1.027)	1.006(0.982,1.030)	1.021(0.997,1.045)	1.007(0.976,1.038)
Males	Time series	1.000(0.985,1.015)	0.997(0.982,1.012)	1.002(0.987,1.016)	1.001(0.983,1.020)	1.008(0.978,1.039)	1.023(0.995,1.053)	1.015(0.987,1.044)	1.022(0.983,1.063)	1.007(0.960,1.056)	1.003(0.957,1.051)	1.019(0.973,1.067)	1.006(0.946,1.070)
	Case-crossover	1.006(0.995,1.017)	1.003(0.992,1.014)	0.976(0.966,0.986)	1.008(0.994,1.022)	1.017(0.995,1.039)	1.035(1.014,1.056)	1.017(0.997,1.037)	1.038(1.010,1.066)	1.016(0.986,1.046)	1.017(0.986,1.048)	1.028(0.999,1.057)	1.028(0.989,1.067)
Females	Time series	0.997(0.978,1.018)	0.999(0.979,1.019)	0.992(0.972,1.012)	1.005(0.980,1.030)	**1.040[a](1.004,1.078)**	1.031(0.994,1.068)	1.009(0.972,1.048)	1.038(0.989,1.090)	0.963(0.905,1.024)	0.965(0.907,1.027)	0.995(0.934,1.059)	0.929(0.857,1.007)
	Case-crossover	1.002(0.987,1.017)	1.009(0.995,1.023)	1.001(0.986,1.016)	1.020(1.001,1.039)	1.036(1.007,1.055)	1.025(0.998,1.052)	1.008(0.982,1.034)	1.045(1.009,1.081)	0.962(0.922,1.002)	0.971(0.932,1.010)	1.002(0.964,1.040)	0.949(0.898,1.000)
<65 years old	Time series	1.000(0.979,1.021)	0.999(0.979,1.020)	1.001(0.981,1.021)	1.001(0.975,1.027)	1.029(0.997,1.062)	1.026(0.995,1.058)	1.004(0.972,1.037)	1.014(0.973,1.057)	0.970(0.914,1.030)	0.972(0.917,1.030)	0.996(0.940,1.056)	0.969(0.900,1.043)
	Case-crossover	1.006(0.983,1.029)	0.991(0.970,1.012)	1.000(0.981,1.019)	0.995(0.968,1.022)	1.039(0.995,1.083)	1.001(0.961,1.041)	0.992(0.954,1.030)	1.007(0.952,1.062)	0.993(0.933,1.053)	0.948(0.891,1.005)	0.990(0.934,1.046)	0.966(0.894,1.038)
≥65 years old	Time series	0.993(0.978,1.008)	0.991(0.977,1.007)	0.997(0.982,1.011)	1.000(0.981,1.018)	1.001(0.966,1.036)	1.020(0.987,1.055)	1.014(0.980,1.048)	1.028(0.980,1.079)	1.024(0.974,1.076)	1.010(0.960,1.063)	1.014(0.965,1.065)	0.992(0.927,1.061)
	Case-crossover	1.003(0.993,1.013)	1.003(0.993,1.013)	1.004(0.995,1.013)	1.014(0.995,1.013)	1.010(0.991,1.029)	1.028(1.010,1.046)	1.016(0.999,1.033)	1.040(1.015,1.065)	1.022(0.996,1.048)	1.023(0.997,1.049)	1.022(0.996,1.048)	1.011(0.977,1.045)
COPD	Time series	0.992(0.972,1.013)	0.989(0.970,1.009)	1.007(0.989,1.025)	1.005(0.981,1.030)	1.008(0.968,1.049)	1.035(0.997,1.074)	1.007(0.969,1.046)	1.030(0.978,1.084)	1.022(0.960,1.088)	0.989(0.929,1.053)	0.966(0.908,1.027)	0.950(0.875,1.031)
	Case-crossover	1.003(0.987,1.019)	0.994(0.978,1.010)	1.010(0.995,1.025)	1.010(0.995,1.025)	1.013(0.982,1.044)	1.032(1.001,1.063)	1.009(0.980,1.038)	1.038(1.000,1.076)	1.025(0.981,1.069)	0.990(0.948,1.032)	0.978(0.937,1.019)	0.973(0.920,1.026)
Pneumonia	Time series	**1.041[a](1.006,1.078)**	**1.054[b](1.018,1.090)**	1.019(0.976,1.063)	**1.057[a](1.005,1.112)**	0.913(0.817,1.021)	**1.090[a](1.001,1.186)**	1.013(0.920,1.114)	1.080(0.952,1.224)	0.971(0.835,1.129)	**0.819[b](0.711,0.944)**	0.942(0.815,1.089)	**0.802[a](0.663,0.970)**
	Case-crossover	1.048(1.007,1.089)	**1.059[a](1.023,1.095)**	1.003(0.968,1.038)	1.061(1.025,1.097)	0.921(0.836,1.006)	1.075(1.001,1.149)	0.965(0.895,1.035)	1.036(0.942,1.130)	0.958(0.859,1.057)	0.828(0.726,0.930)	0.930(0.833,1.027)	0.787(0.669,0.905)
Asthma	Time series	0.992(0.967,1.018)	**0.965[a](0.938,0.993)**	**0.965[a](0.938,0.992)**	**0.951[b](0.918,0.985)**	**1.050[a](1.007,1.095)**	1.019(0.974,1.066)	1.037(0.992,1.085)	1.050(0.989,1.113)	0.972(0.905,1.045)	1.073(0.994,1.159)	1.056(0.978,1.140)	1.055(0.960,1.160)
	Case-crossover	0.994(0.972,1.016)	0.962(0.941,0.983)	0.963(0.941,0.985)	0.960(0.934,0.986)	1.034(0.993,1.075)	1.004(0.966,1.042)	1.014(0.976,1.052)	1.042(0.995,1.089)	0.977(0.920,1.034)	1.064(1.007,1.121)	1.037(0.983,1.091)	1.069(1.001,1.137)

a:The RRs or ORs are statistically significant (P-value <0.05).b: The RRs or ORs are statistically significant (P-value <0.01)
